# H3K36 Trimethylation-Mediated Epigenetic Regulation is Activated by Bam and Promotes Germ Cell Differentiation During Early Oogenesis in *Drosophila*

**DOI:** 10.1242/bio.201410850

**Published:** 2015-01-08

**Authors:** Masanori Mukai, Seiji Hira, Katsuhiro Nakamura, Shoichi Nakamura, Hiroshi Kimura, Masanao Sato, Satoru Kobayashi

**Affiliations:** 1 Department of Biology, Faculty of Science and Engineering, Konan University, Okamoto, Higashinada, Kobe 658-8501, Japan; 2 Graduate School of Natural Science, Konan University, Okamoto, Higashinada, Kobe 658-8501, Japan; 3 Institute for Integrative Neurobiology, Konan University, Okamoto, Higashinada, Kobe 658-8501, Japan; 4 Research Fellow of Japan Society for the Promotion of Science, Tokyo 102-0083, Japan; 5 Department of Biological Sciences, Graduate School of Bioscience and Biotechnology, Tokyo Institute of Technology, Yokohama, 226-8501, Japan; 6 Okazaki Institute for Integrative Bioscience, National Institute for Basic Biology, National Institutes of Natural Sciences, Higashiyama, Myodaiji, Okazaki 444-8787, Japan; 7 Life Science Center, Tsukuba Advanced Research Alliance, University of Tsukuba, Tsukuba, Ibaraki 305-8577, Japan

**Keywords:** *Drosophila*, Germ cell, Differentiation factor, Histone modification, Transcriptional control

## Abstract

Epigenetic silencing is critical for maintaining germline stem cells in *Drosophila* ovaries. However, it remains unclear how the differentiation factor, Bag-of-marbles (Bam), counteracts transcriptional silencing. We found that the trimethylation of lysine 36 on histone H3 (H3K36me3), a modification that is associated with gene activation, is enhanced in Bam-expressing cells. H3K36me3 levels were reduced in flies deficient in Bam. Inactivation of the Set2 methyltransferase, which confers the H3K36me3 modification, in germline cells markedly reduced H3K36me3 and impaired differentiation. Genetic analyses revealed that Set2 acts downstream of Bam. Furthermore, *orb* expression, which is required for germ cell differentiation, was activated by Set2, probably through direct H3K36me3 modification of the *orb* locus. Our data indicate that H3K36me3-mediated epigenetic regulation is activated by *bam*, and that this modification facilitates germ cell differentiation, probably through transcriptional activation. This work provides a novel link between Bam and epigenetic transcriptional control.

## INTRODUCTION

Post-translational modifications to core histone proteins are proposed to regulate essential cellular functions, including transcriptional activation and repression. For instance, histone H3 methylations at lysine 4 (H3K4) and at lysine 36 (H3K36) are usually associated with gene activation, whereas methylations of lysine 9 (H3K9) and lysine 27 (H3K27) are associated with gene repression. Several histone modifications play fundamental roles in the maintenance of embryonic stem cells, particularly with respect to their developmental potential (Bloushtain-Qimron et al., 2009; Jiang et al., 2011); histone modifications are also associated with the maintenance of stem cells in adult tissues (Buszczak et al., 2009).

In the adult *Drosophila* ovary, the germline stem cells (GSCs) at the tip of the germaria are maintained in their niche. After GSC division, the daughter cell that is displaced from the niche becomes a cystoblast, and subsequently differentiates into a 16-cell cyst interconnected by the branched fusome; 1 germ cell develops into the oocyte and the other 15 germ cells form nurse cells. The bone morphogenic protein (BMP)-like molecules produced from the niche maintain GSCs by directly repressing *bag-of-marbles* (*bam*), which encodes a key differentiation factor (Ohlstein and McKearin, 1997; Chen and McKearin, 2003b). When a cystoblast exits the niche, the Bam produced in the cystoblast antagonizes the Nanos/Pumilio translational repressor complex to promote differentiation (Li et al., 2009). In addition to BMP signalling, epigenetic silencing is essential for GSC maintenance. The functions of *scrawny* and *eggless*, both of which encode histone-modifying enzymes that are associated with gene silencing, are required for GSC maintenance (Buszczak et al., 2009; Wang et al., 2011). However, the mechanisms by which epigenetic regulation promotes differentiation, and by which Bam counteracts gene silencing remain unclear. We found that the levels of trimethylation of H3K36 (H3K36me3) in cystoblasts were enhanced by Set2 methyltransferase. Set2 acted downstream of *bam* and promoted differentiation. Furthermore, Set2 activated *orb* expression, which is required for cyst differentiation. Our results indicate that H3K36me3 in cystoblasts is developmentally controlled by *bam*, and that this modification facilitates cystoblast differentiation, probably through transcriptional activation.

## MATERIALS AND METHODS

### Fly stocks

The wild-type strain used was *Oregon-R*. *Set2^1^/FM7* was a gift from Dr. M. Kuroda. *bam^86^/TM3* was a gift from Dr. D. M. McKearin. *orb^dec^/+*, *UAS-Set2.IR*, *v^24^ P{FRT}101, P*{*ubi-GFP FRT101}, P{MKRS, hs*-*FLP 86E}*, and *P{hs-Gal4}* were obtained from the Bloomington Stock Center. *A2BP1^KG06463^*/+ was obtained from the Drosophila Genetic Resource Center. *UAS-Set2 RNAi* (106459) was obtained from the Vienna Drosophila RNAi Center (VDRC). All stocks were maintained at 25°C or at room temperature in standard *Drosophila* medium unless otherwise noted.

### Immunohistochemistry

Immunostaining was carried out as described (Mukai et al., 2011). Monoclonal antibodies specific for H3K4me1 (CMA301), H3K4me2 (CMA302), H3K4me3 (CMA303), H3K27ac (CMA309), H3K27me3 (140-20 IE7) and H3K36me3 (144-6 13C9) monoclonal antibodies were used at a 1:10 dilution (Kimura et al., 2008). The following primary antibodies were used: rabbit anti-Vas antibody (1:500), mouse anti-Set2/dHypb (1:15) (Bell et al., 2007) and rabbit anti-GFP (1:200, Invitrogen). Monoclonal antibodies obtained from the Developmental Studies Hybridoma Bank included mouse anti-1B1 (1:10), mouse anti-Orb 4H8 (1:30) and rat anti-DN-cadherin (1:20). Alexa Fluor 488– and Alexa Fluor 568–conjugated secondary antibodies (Molecular Probes) were used at 1:1000. Stained ovaries were observed by confocal microscopy (TCS NT, Leica Microsystems). Optical sections taken at 1 µm intervals with a picture size of 512×512 pixels were processed using Adobe Photoshop CS3 and CS6.

### Phenotypic analysis of ovaries

Clones of mutant cells were generated by FLP-mediated mitotic recombination as described (Mukai et al., 2011). We introduced *Set2^1^* into the chromosome carrying FRT by meiotic recombination. We generated *Set2^−^* germline clones by using the *Set2^1^* FRT chromosome. Control germline clones were generated using FRT chromosomes without the mutation. For RNAi knockdown of *Set2*, *nanos-Gal4*/+; *UAS-Set2.IR* /+ females were cultured at 30°C up to adulthood. *nanos-Gal4/+* females raised at 30°C served as controls. Ovaries were processed for immunostaining. We also used an independent *UAS-Set2* RNAi line (106459), and found that the expression of *Set2* RNAi elicited similar defects in cyst formation. To examine the effect of ectopic *bam* expression on GSC differentiation, *hs-bam* flies were heat-shocked for 1 hour at 37°C, transferred to vials at 25°C for 1 hour, and heat-shocked again at 37°C for 1 hour, and then cultured at 25°C for the indicated period prior to dissection. The intracellular localization of Set2 in germ cells was affected in germaria from wild-type females heat-shocked as described above (data not shown). Thus, we induced *hs-bam* expression under mild conditions. To examine the effect of ectopic *bam* expression on Set2 nuclear localization, *hs-bam* and wild-type flies were cultured at 30°C for 3 days, and then the flies were used for immunostaining.

### Chromatin immunoprecipitation (ChIP) assay

A ChIP assay was performed using the wild-type and *bam^86^* mutant ovaries as described (Baxley et al., 2011). Immunoprecipitation was performed using 1 µg of antibody. As a control, normal mouse IgG (Jackson ImmunoResearch Laboratories) was used. Anti-H3K36me3, anti-H3K4me3 (Kimura et al., 2008), and anti-RNA polymerase II (8WG16; Covance) antibodies were used for the ChIP assays. Input DNA, mock-precipitated DNA, and DNA from the ChIP assays were analyzed by PCR. Quantitative PCR analyses were performed using GeneAce SYBR qPCR Mix (Nippon Gene). The sequences of the primers used for the ChIP assays are listed in supplementary material Table S1.

## RESULTS AND DISCUSSION

### H3K36me3 is associated with cystoblast differentiation

**Fig. 1.**
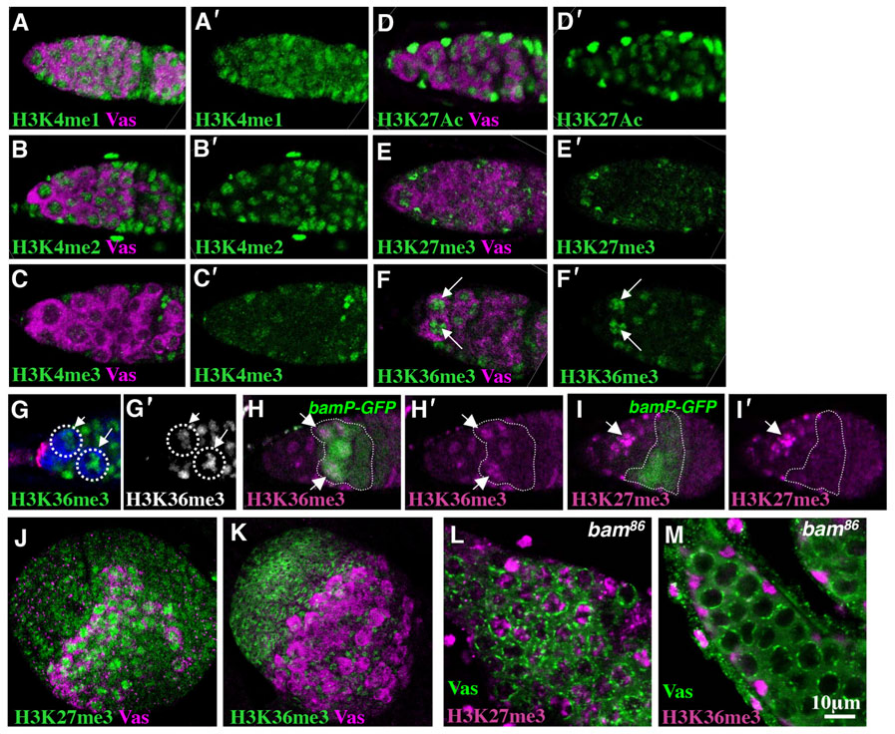
H3K36me3 is upregulated in differentiating cystoblasts. (A–F) Wild-type ovarioles were double-stained for the indicated histone modifications (green), and for Vas (magenta), a germ cell marker. (A′–F′) Histone modification channel is shown separately. Strong H3K36me3 signals are shown in the cystoblasts (F, arrows). (G) An ovariole triple-stained for H3K36me3 (green), Vas (blue) and DN-cadherin (red), which labels the GSC niche. (G′) H3K36me3 channel is shown alone. Stronger H3K36me3 signals are detected in the cystoblast (arrow) than in the GSC (arrowhead). (H,I) *bamP-GFP* ovarioles were double-stained for H3K36me3 (H, magenta) or for H3K27me3 (I, magenta) and GFP (green). (H′) H3K36me3 channel is shown alone. (I′) H3K27me3 channel is shown alone. (J,K) Ovaries from 3rd instar larvae were double-stained for H3K27me3 (J, green) or H3K36me3 (K, green) and Vas (magenta). (L,M) *bam^86^*mutant ovarioles were double-stained for H3K27me3 (L, magenta) or H3K36me3 (M, magenta) and Vas (green).

To examine histone modifications in differentiating germ cells, we stained wild-type ovaries using monoclonal antibodies specific for histone modifications (Fig. 1; Kimura et al., 2008). We found that the H3K36me3 histone modification associated with active genes accumulated in differentiating cystoblasts (Fig. 1F,G). H3K36me3 signals were increased in the differentiating cystoblasts that expressed the *bam* reporter gene (*bam-GFP*; Chen and McKearin, 2003a) (Fig. 1H). By contrast, the H3K27me3 modification associated with gene repression accumulated in early germ cells, and its signals decreased as the cells differentiated (Fig. 1E,I). These results suggest that the H3K36me3 levels were upregulated in differentiating cystoblasts. Next, we examined H3K36me3 levels in the ovaries of the third instar larvae and *bam^86^* mutant adult females, both of which contain undifferentiated germ cells. Although H3K27me3 signals were detected in these undifferentiated germ cells, we did not detect strong H3K36me3 signals (Fig. 1J–M). Taken together, these data supported the idea that H3K36me3-mediated epigenetic regulation may be involved in germ cell differentiation.

### Set2 is required for both H3K36me3 accumulation and cyst formation

**Fig. 2.**
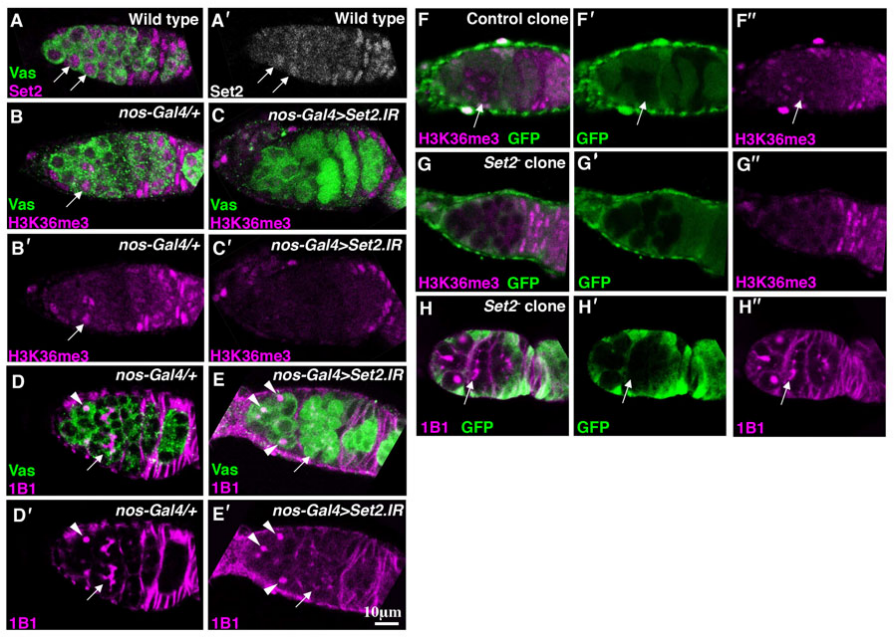
Set2 is required for H3K36me3 accumulation and cyst formation. (A) An ovariole was double-stained for Set2 (magenta) and Vas (green). (A′) Set2 channel is shown alone. Nuclear Set2 levels increased in differentiating cystoblasts (arrows). (B,C) Control (*nos-Gal4/+*) (B) and *nos-Gal4>UAS-Set2.IR* (C) ovarioles were double-stained for H3K36me3 (magenta) and Vas (green). (B′,C′) H3K36me3 channel is shown separately. (D,E) *nos-Gal4/+* (D) and *nos-Gal4>UAS-Set2.IR* (E) ovarioles were double-stained for 1B1 (magenta), which labels spectrosome (arrowheads) and fusome (arrows), and Vas (green). (D′,E′) 1B1 channel is shown separately. (F–H) Ovarioles containing control (F) and *Set2*^−^ clones (G,H) were double-stained for H3K36me3 (F,G, magenta), or 1B1 (H, magenta) and GFP (green). (F′–H′) GFP channel is shown separately. (F″,G″) H3K36me3 channel is shown separately. (H″) 1B1 channel is shown alone. An absence of GFP marks the clones. The arrow in H indicates fragmented fusomes.

Set2 methyltransferase is responsible for the H3K36me3 modification (Larschan et al., 2007; Stabell et al., 2007). Immunostaining revealed that, in the germarium region, Set2 was expressed in most of the germline cells, and that nuclear Set2 levels increased in differentiating cystoblasts (Fig. 2A). To determine whether Set2 participates in H3K36me3 accumulation and differentiation, we inhibited Set2 expression by using an *UAS-Set2.IR* line (Stabell et al., 2007). Set2 levels in germ cells were reduced by the expression of *Set2* RNAi (supplementary material Fig. S1). Specifically, while Set2 signals in differentiating cystoblasts were detected in 100% of control (*nanos-Gal4/+*) germaria (*n* = 97), the Set2 signals in the cystoblasts were significantly reduced in 57% of the germaria, when *Set2* RNAi was expressed in germ cells under the control of the *nanos-Gal4* driver (*n* = 170; supplementary material Fig. S1). Next, we investigated H3K36me3 levels in the ovaries expressing *Set2* RNAi. As expected, H3K36me3 levels were reduced as a consequence of *Set2* RNAi treatment. In control ovaries, H3K36me3 signals in differentiating cystoblasts were detected in 97% of germaria (Fig. 2B; *n* = 30). By contrast, when *Set2* RNAi was expressed in germ cells under the control of the *nanos-Gal4* driver, H3K36me3 signals in cystoblasts were severely reduced in 41% of the germaria (Fig. 2C; *n* = 39, *P*<0.001). Moreover, germ cell differentiation was impaired because of the expression of *Set2* RNAi. In 96% of the control germaria, cysts with branched fusomes were observed (Fig. 2D; *n* = 47). However, fragmented fusomes were detected in 34% of the germaria expressing *Set2* RNAi (Fig. 2E; *n* = 67; *P*<0.001). These results indicate that Set2 was required for both H3K36me3 accumulation and cyst formation. We next performed mosaic analysis by using a *Set2* null allele *Set2^1^* (Larschan et al., 2007). Strong H3K36me3 signals were observed in 80% of the control germline clones (Fig. 2F; *n* = 30). By contrast, H3K36me3 levels were considerably reduced in 74% of the *Set2^−^* cystoblasts (Fig. 2G; *n* = 38). Furthermore, we observed a differentiation defect similar to that induced by *Set2* RNAi treatment in 84% of *Set2^−^* mutant cysts (Fig. 2H; *n* = 37). These results suggest that Set2 is intrinsically required both for H3K36me3 accumulation in cystoblasts and for differentiation.

### Set2 acts downstream of *bam*

**Fig. 3.**
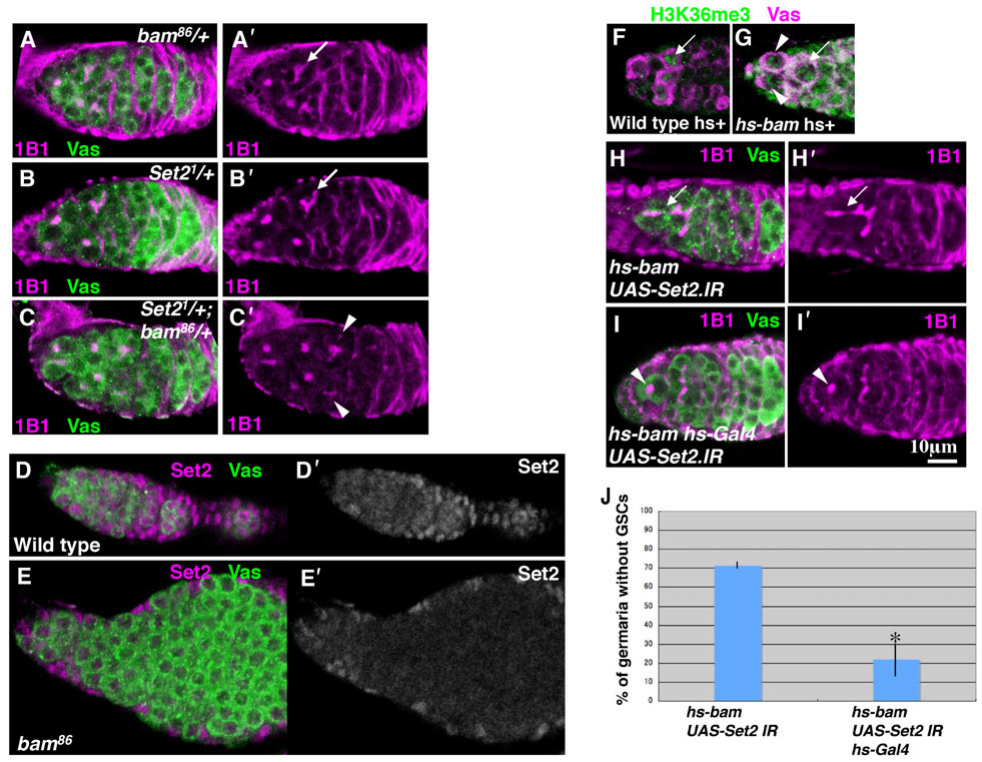
*Set2* genetically interacts with *bam*. (A–C) Ovarioles from *bam^86^/+*(A), *Set2^1^/+* (B) and *Set2^1^/+; bam^86^/+* (C) were double-stained for 1B1 (magenta) and Vas (green). (A′–C′) 1B1 channel is shown separately. Arrows (A′,B′) indicate branched fusomes. Arrowheads (C′) indicate fragmented fusomes. (D,E) Wild-type (D) and *bam^86^*mutant (E) ovarioles were double-stained for Set2 (magenta) and Vas (Green). (D′,E′) Set2 channel is shown alone. (F,G) Ovarioles from heat-shocked wild-type (F) and *hs-bam* females (G) were double-stained for H3K36me3 (green) and for Vas (magenta). Strong H3K36me3 signals in GSCs are shown in the *hs-bam* ovariole (arrowheads). (H,I) Ovarioles from 24 hours PHS *hs-bam, UAS-Set2.IR* (H) and *hs-bam, UAS-Set2.IR, hs-Gal4* (I) flies were double-stained for 1B1 (magenta) and Vas (green). (H′,I′) 1B1 channel is shown separately. While a cyst occupies the niche in the ovariole expressing *hs-bam* (H, arrow), a GSC is found in the ovariole expressing both *hs-bam* and *Set2* RNAi (I, arrowhead). (J) The GSC loss of phenotype induced by *bam* is suppressed by *Set2* RNAi. Data represent the mean±s.d. *P<0.02.

To investigate the potential regulatory link between Set2 and Bam, we analyzed their genetic interaction. Reduction in *Set2* activity by introduction of a single copy of *Set2^1^* dominantly increased the number of germaria with the differentiation defect in *bam^86^/+* flies (Fig. 3A–C). Fragmented fusomes were observed in 26% of germaria from the *Set2^1^/+*; *bam^86^/+* females (*n* = 125), as compared to 5% in *bam^86^/+* (*n* = 77) and 3% in *Set2^1^/+* (*n* = 68) females. These results indicated that *Set2* cooperates with *bam* to promote cyst formation. To determine whether *bam* expression requires *Set2* activity, we examined Bam expression in *Set2^−^* germline clones by immunostaining. Indeed, Set2 activity in germ cells was dispensable for *bam* expression (supplementary material Fig. S2). Conversely, nuclear Set2 expression in the germ cells was significantly reduced by *bam* mutation, suggesting that *bam* is involved in the regulation of Set2 in these cells (Fig. 3D,E). This result is consistent with the observation that H3K36me3 levels were reduced by *bam* mutation. Moreover, reducing of *bam* activity by introducing of a single copy of *bam^86^* dominantly increased the number of germaria with weaker H3K36me3 signals in *Set2^1^/+* flies. Decreased H3K36me3 signals in the cystoblasts were observed in 29% of germaria from the *Set2^1^/+; bam^86^/+* females (*n* = 157), as compared to 3% in *Set2^1^/+* (*n* = 117) and 2% in *bam^86^/+* (*n* = 134) females (supplementary material Fig. S3). These data prompted us to explore the mechanism of regulation of Set2 activity by *bam*.

To address whether *bam* is sufficient for H3K36me3 accumulation, we examined H3K36me3 levels in the ovaries carrying the *hs*-*bam* transgene, which is used to ectopically express *bam+* by heat shock treatment (Ohlstein and McKearin, 1997). No GSCs with a strong H3K36me3 signal were observed in germaria from wild-type females 1 hour post-heat shock (PHS; *n* = 42). However, H3K36me3 levels in the GSCs were significantly increased in 51% of the germaria from *hs-bam* females 1 hour PHS (Fig. 3F,G; *n* = 65), indicating that ectopic *bam* expression is sufficient for H3K36me3 accumulation. Because Set2 is responsible for H3K36me3, we speculated that *bam* may regulate Set2 activity to control H3K36me3 accumulation and GSC differentiation. To determine whether Set2 activity is required for these *bam*-mediated processes, we examined the effect of a reduction in Set2 activity on the GSC differentiation induced by *bam*. When *bam+* was ectopically expressed by heat shock, GSC differentiation was induced as previously reported (Ohlstein and McKearin, 1997). In 71% of ovaries from *hs-bam* flies dissected 24 hours PHS, we found that differentiating cysts, instead of GSCs, occupied the tip of germaria (*n* = 79; Fig. 3H). By contrast, when both *bam* and *Set2* RNAi were ectopically expressed, GSC loss was significantly suppressed (19.6%, *n* = 189; *P*<0.02) (Fig. 3I,J). These data suggest that Set2 activity is regulated by Bam, and that Set2 acts downstream of *bam* and promotes differentiation.

We found that nuclear Set2 levels were increased in differentiating cystoblasts (Fig. 2A). Furthermore, nuclear Set2 levels in germ cells were reduced by *bam* mutation (Fig. 3E). We speculated that *bam* may regulate Set2 nuclear localization. Therefore, we examined whether *bam* expression is sufficient for Set2 nuclear accumulation. We investigated the subcellular localization of Set2 in *hs-bam* flies cultured at 30°C (see Materials and Methods). First, we examined H3K36me3 levels in the GSCs. H3K36me3 levels in GSCs were increased in 36% of the germaria from the *hs-bam* females (*n* = 84), as compared to 6% in wild-type females (*n* = 79, *P*<0.01; supplementary material Fig. S4A,B). This result suggests that the ectopic expression of *bam* is sufficient for H3K36me3 accumulation. Next, we investigated Set2 subcellular localization in GSCs of *hs-bam* females cultured at 30°C. Nuclear Set2 levels in GSCs were increased in 54% of the germaria from the *hs-bam* females (*n* = 65), as compared to 12% in wild-type females (*n* = 79, *P*<0.01; supplementary material Fig. S4C,D). These results suggest that *bam* promotes the nuclear accumulation of Set2.

### *Set2* function is required for the proper activation of *orb* expression in cysts

**Fig. 4.**
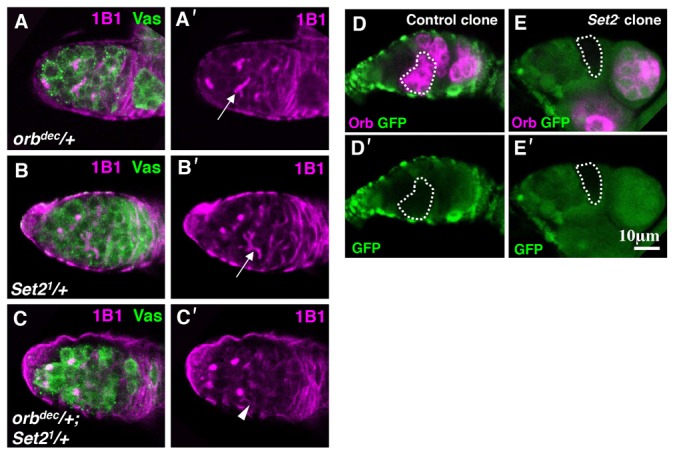
*Set2* is required for the proper activation of *orb* expression in cysts. (A–C) Ovarioles from *orb^dec^/+* (A), *Set2^1^/+* (B) and *Set2^1^/+; orb^dec^/+* (C) were double-stained for 1B1 (magenta) and Vas (green). (A′–C′) 1B1 channel is shown separately. Arrows (A′,B′) indicate branched fusomes. An arrowhead (C′) indicates fragmented fusomes. (D,E) Ovarioles containing control (D) and *Set2*^−^ clones (E) were double-stained for Orb (magenta) and GFP (green). (D′,E′) GFP channel is shown alone. The Orb signal is reduced in the *Set2*^−^ cyst (dotted line in E).

**Fig. 5.**
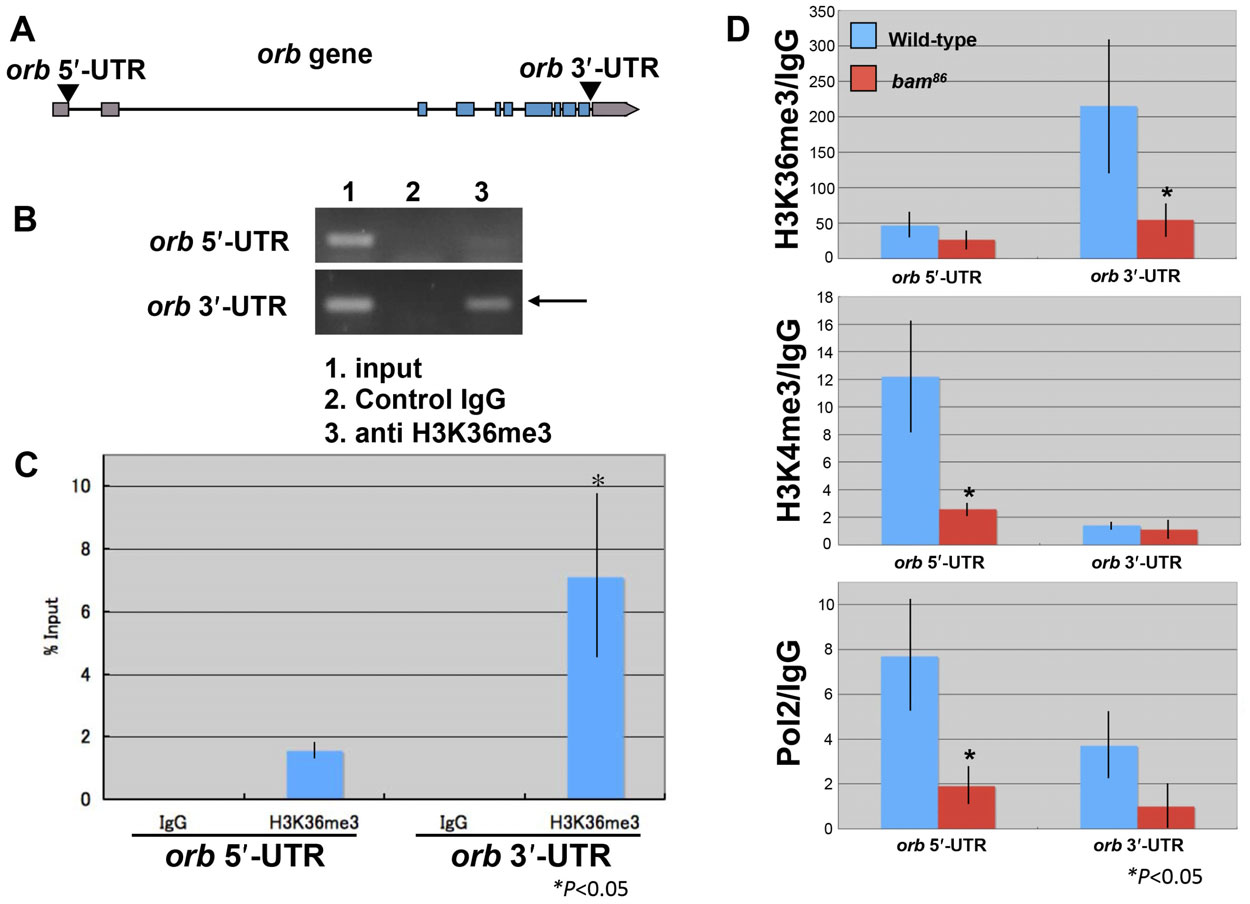
*bam* is required for H3K36me3 enrichment in the 3′-UTR region of *orb*. (A) Schematic representation of the *orb* locus. (B,C) The H3K36me3 modification is detected in the 5′- and 3′-UTRs of the *orb* gene by PCR (B) and quantitative real-time PCR (C). (C) Wild-type ovaries were used for a ChIP assay. Input DNA, mock-precipitated DNA, and DNA from the ChIP assay were analyzed by quantitative real-time PCR. Percent input was calculated by using input as standards. Data represent the mean ± s.d. The significance was calculated by comparing the values detected at the 5′- or 3′-UTRs (*P<0.05; analysis of variance). (D) The levels of H3K36me3 and H3K4me3 modifications and RNA polymerase II (Pol2) detected in the *orb* gene 5′- and 3′-UTRs by quantitative real-time PCR. Ovaries dissected from wild-type and *bam^86^* mutant flies were used for the ChIP assay. The values are expressed as a fold increase relative to the IgG control. The significance was calculated by comparing the values obtained using wild-type and *bam* mutant ovaries (*P<0.05; analysis of variance). All ChIP assays were performed in 3 biological replicates.

To understand the mechanism by which Set2 regulates germ cell differentiation, we analyzed the genetic interaction between *Set2* and the differentiation genes *A2BP1* and *orb*, both of which are required for cyst differentiation (Lantz et al., 1994; Tastan et al., 2010). Reduction of *Set2* activity by introduction of a single dose of *Set2^1^* dominantly increased the number of germaria exhibiting a differentiation defect in *orb^dec^/+* flies (Fig. 4A–C). In 24% of germaria from the *Set2^1^/+*; *orb^dec^/+* females, fragmented fusomes were observed (*n* = 132), as compared with 4% in *orb^dec^/+* (*n* = 73) and 7% in *Set2^1^/+* females (*n* = 106). By contrast, the reduction of *Set2* activity did not significantly affect cyst formation in *A2BP1^KG06463^*/+ ovaries (data not shown). These results implied that *Set2* function is required to specifically regulate *orb* expression and promote cyst formation. To confirm this, we examined *orb* expression in *Set2^−^* cyst clones. Deletion of *Set2* led to the delayed activation of *orb*. Although 74% of the control cyst clones located at the boundary of germarium regions 1 and 2a initiated *orb* expression (*n* = 50), only 31% of *Set2^−^* cyst clones expressed *orb* (Fig. 4D,E; *n* = 62, *P*<0.001). Most (61%) of the *Set2^−^* cyst clones in germarium region 2b recovered *orb* expression (*n* = 62). These observations suggest that *Set2* was required for the proper activation of *orb* in differentiating cysts. Next, we investigated the H3K36me3 state of the *orb* locus in the ovaries. ChIP assays demonstrated that the H3K36me3 enrichment in the 3′-UTR region of *orb* was significantly higher than in the 5′-UTR region (Fig. 5B–D). It has been reported that the H3K36me3 modification exhibits a 3′-bias, such that H3K36me3 is preferentially enriched at the 3′ regions of actively transcribed genes (Larschan et al., 2007; Barski et al., 2007). Our results support the idea that *orb* expression in differentiating cysts is controlled in part by H3K36me3-mediated epigenetic regulation.

Next, we investigated the H3K36me3 status in the *orb* gene in *bam^86^* mutant ovaries. ChIP assays showed that *bam* mutation reduced the amount of H3K36me3 in the 3′-UTR region of the *orb* gene (Fig. 5D). The H3K36me3 modification is linked to transcriptional elongation (Krogan et al., 2003). Therefore, our results suggested that *bam* activates *orb* expression through the epigenetic control. Additionally, H3K4me3 and RNA polymerase II levels in the 5′-UTR region of the *orb* gene were also reduced by *bam* mutation (Fig. 5D), implying a role for *bam* in transcriptional initiation. To investigate this possibility, further investigation will be needed in order to identify the enzymes responsible for H3K4me3 and exploring the interactions between *bam* and those enzymes.

Our results showed that H3K36me3 levels are regulated by *bam*. As a cytoplasmic protein, Bam may indirectly regulate Set2 nuclear localization. Set2 exerts its functions through the interactions with cofactors (Fuchs et al., 2012). Understanding the mechanism by which Bam regulates Set2 will require the identification of the cofactors that mediate the nuclear transport of Set2. Our data suggest a link between Bam and epigenetic transcriptional control. Bam may counteract epigenetic silencing in GSCs through H3K36me3-mediated epigenetic regulation. We show that *orb* expression is activated by epigenetic regulation. Because *orb* encodes a cytoplasmic polyadenylation element-binding protein, Orb may control translation in differentiating cysts in a polyadenylation-associated manner. Bam antagonizes the Nanos/Pumilio complex, which suppresses the translation of target mRNAs that encode differentiation factors (Li et al., 2009). However, the identity of the target mRNAs and the mechanisms for transcriptional activation have not yet been elucidated. Because Set2 is required for *bam*-induced GSC differentiation, studies focused on identifying the genes marked by H3K36me3 and on their epigenetic regulation will aid in the identification of the differentiation genes. Because Set2 is linked to transcriptional elongation (Krogan et al., 2003), differentiation genes in GSCs might be poised for expression, but may be kept awaiting *bam* expression for full activation. We anticipate that our results will facilitate a better understanding of the epigenetic mechanisms that regulate gametogenesis.

## Supplementary Material

Supplementary Material
