# Supplementary material for: H3K36 Trimethylation-Mediated Epigenetic Regulation is Activated by Bam and Promotes Germ Cell Differentiation During Early Oogenesis in Drosophila
Source: Biol Open. 2015 Jan 8;4(2):119–24. doi: 10.1242/bio.201410850 (PMC4365480; doi:10.1242/bio.201410850)
Supplement: Supplementary Material [file supp_4_2_119__index.html]

H3K36 Trimethylation-Mediated Epigenetic Regulation is Activated by Bam and Promotes Germ Cell Differentiation During Early Oogenesis in Drosophila — H3K36 Trimethylation-Mediated Epigenetic Regulation is Activated by Bam and Promotes Germ Cell Differentiation During Early Oogenesis in Drosophila — Supplementary Material 

# H3K36 Trimethylation-Mediated Epigenetic Regulation is Activated by Bam and Promotes Germ Cell Differentiation During Early Oogenesis in *Drosophila*

## bio.201410850 Supplementary Material

**Files in this Data Supplement:**

- Supplementary Material - Masanori Mukai et al. doi: 10.1242/bio.201410850
