# Supplementary material for: H3K36 Trimethylation-Mediated Epigenetic Regulation is Activated by Bam and Promotes Germ Cell Differentiation During Early Oogenesis in Drosophila
Source: Biol Open. 2015 Jan 8;4(2):119–24. doi: 10.1242/bio.201410850 (PMC4365480; doi:10.1242/bio.201410850)
Supplement: Supplementary Material [file supp_bio.201410850_bio.201410850-s1.pdf]

Biology Open (2015) 000, 1–6 doi:10.1242/bio.201410850

Supplementary Material

Masanori Mukai et al. doi: 10.1242/bio.201410850

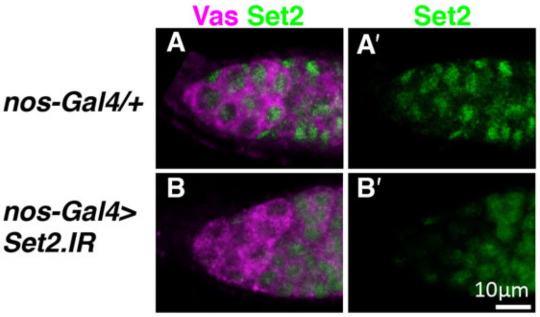

Fig. S1. RNAi-mediated knockdown of Set2 in cystoblasts. (A,B) Ovarioles from *nos-Gal4/+* (A) and *nos-Gal4>UAS-Set2.IR* (B) females were double-stained for Vas (magenta) and Set2 (green). (A′,B′) Set2 channel is shown alone. Set2 levels in cystoblasts are reduced in the germarium expressing *Set2* RNAi.

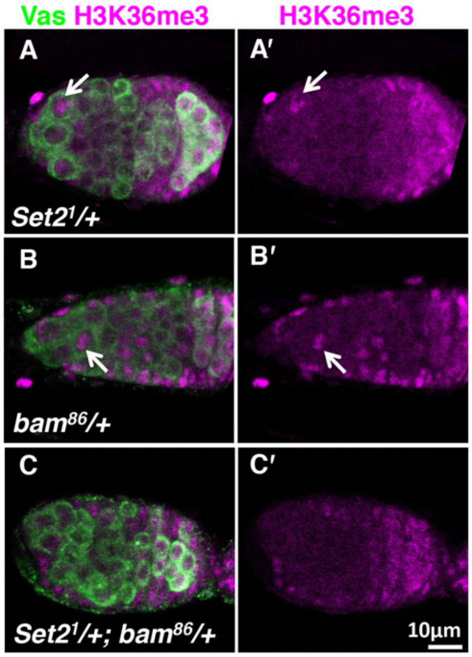

Fig. S3. Reduction in *bam* activity decreases H3K36me3 levels in the germ cells of *Set2*^{1}/+ ovaries. Ovarioles from *Set2*^{1}/+ (A), *bam*^{86}/+ (B) and *Set2*^{1}/+; *bam*^{86}/+ (C) were double-stained for H3K36me3 (magenta) and Vas (green). (A′–C′) H3K36me3 channel is shown separately.

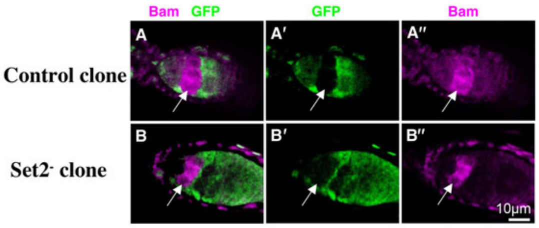

Fig. S2. *Set2* function in germ cells is dispensable for Bam expression. (A,B) Ovarioles containing control (A) and *Set2*$^{-}$ clones (B) were double-stained for Bam (magenta) and GFP (green). (A′,B′) GFP channel is shown alone. (A″,B″) Bam channel is shown separately. Bam is expressed in the *Set2*$^{-}$ cyst (arrow in B).

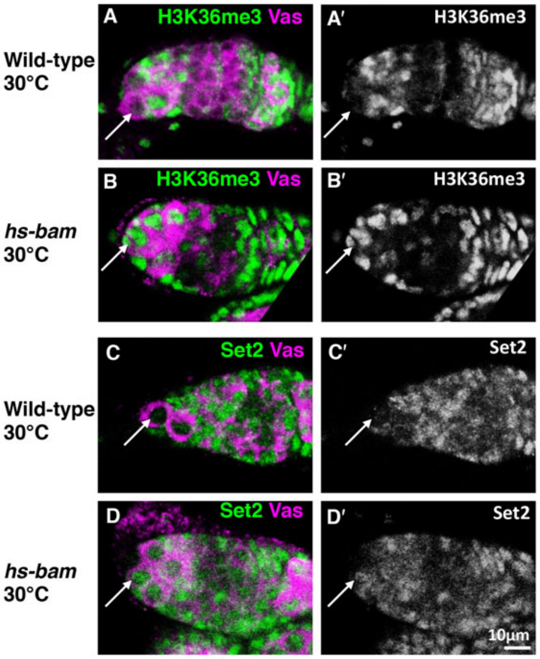

Fig. S4. Ectopic *bam* expression increases nuclear Set2 levels in GSCs. (A,B) Ovarioles from wild-type (A) and *hs-bam* (B) flies cultured at 30˚C were double-stained for H3K36me3 (green) and Vas (magenta). (A′,B′) H3K36me3 channel is shown separately. (C,D) Ovarioles from wild-type (C) and *hs-bam* (D) flies cultured at 30˚C were double-stained for Set2 (green) and Vas (magenta). (C′,D′) Set2 channel is shown separately. Arrows indicate GSCs.

Table S1. ChIP primers

	ChIP primers
orb 5′-UTR	For: 5′-GAGTAGTTTGGCGAAACAGCATCG-3′
	Rev:5′-CGCACTTGCAATCGCATCCCGT-3′
orb 3′-UTR	For: 5′-GTCAACAGCATCAGGCGATC-3′
	Rev: 5′-CAATGATAATGACGATGATGAGCCC-3′
